# Investigation of the best effective fold of data augmentation for training deep learning models for recognition of contiguity between mandibular third molar and inferior alveolar canal on panoramic radiographs

**DOI:** 10.1007/s00784-023-04992-6

**Published:** 2023-04-12

**Authors:** Dhanaporn Papasratorn, Suchaya Pornprasertsuk-Damrongsri, Suraphong Yuma, Warangkana Weerawanich

**Affiliations:** 1 Department of Oral and Maxillofacial Radiology, Faculty of Dentistry, Mahidol University, 6, Yothi Road, Ratchathewi District, Bangkok, 10400 Thailand; 2 Department of Physics, Faculty of Science, Mahidol University, 272 Rama VI Road, Ratchathewi District, Bangkok, 10400 Thailand

**Keywords:** Deep learning, Inferior alveolar canal, Panoramic radiography, Third molar

## Abstract

**Objectives:**

This study aimed to train deep learning models for recognition of contiguity between the mandibular third molar (M3M) and inferior alveolar canal using panoramic radiographs and to investigate the best effective fold of data augmentation.

**Materials and methods:**

The total of 1800 M3M cropped images were classified evenly into contact and no-contact. The contact group was confirmed with CBCT images. The models were trained from three pretrained models: AlexNet, VGG-16, and GoogLeNet. Each pretrained model was trained with the original cropped panoramic radiographs. Then the training images were increased fivefold, tenfold, 15-fold, and 20-fold using data augmentation to train additional models. The area under the receiver operating characteristic curve (AUC) of the 15 models were evaluated.

**Results:**

All models recognized contiguity with AUC from 0.951 to 0.996. Ten-fold augmentation showed the highest AUC in all pretrained models; however, no significant difference with other folds were found. VGG-16 showed the best performance among pretrained models trained at the same fold of augmentation. Data augmentation provided statistically significant improvement in performance of AlexNet and GoogLeNet models, while VGG-16 remained unchanged.

**Conclusions:**

Based on our images, all models performed efficiently with high AUC, particularly VGG-16. Ten-fold augmentation showed the highest AUC by all pretrained models. VGG-16 showed promising potential when training with only original images.

**Clinical relevance:**

Ten-fold augmentation may help improve deep learning models’ performances. The variety of original data and the accuracy of labels are essential to train a high-performance model.

## Introduction

The removal of the impacted teeth, especially the mandibular third molars (M3Ms), is one of the most common dental surgeries. One important structure near the M3M is the inferior alveolar canal (IAC) which contains the inferior alveolar nerve (IAN). Damaging the IAN during M3M removal process may lead to permanent neurosensory impairments of the innervated areas, including lower anterior teeth, lower lip, and chin, affecting patients’ quality of life [1].

Two-dimensional panoramic radiography is a common pre-operative technique to assess the close proximity between M3M and IAC [2,3]. Darkening of the root [4–7], narrowing of the root [4], interruption of the white line [5–8], and diversion of the IAC [4,7] are examples of risk signs spotted in the panoramic radiograph. The risk signs can be subjective among observers and difficult for inexperienced dentists to observe [9]. This could impact the referral decision by the newly graduated dentist who could be the only dentist positioned in the rural hospital of developing countries.

Three-dimensional images obtained from cone beam computed tomography (CBCT) can distinctively show a clear view of the position and proximity of the M3M and the IAC [6,8,10–12]. However, this comes at the price of high costs and unavailability in rural hospitals. Recently, machine learning has been widely applied to the assessment of medical images due to its ability to learn and extract image features without being specifically programmed [13–15]. One of the most notable machine learning methods is the application of deep learning (DL) models using convolutional neural networks (CNNs), which have shown high diagnostic performance in analyzing radiographic images [16].

DL has been applied in the detection, classification, and segmentation of the M3M and the IAC. The results showed that DL is highly potential to be used in the clinical workflow [17]. The diagnostic performance of three different DL models was studied to classify the relationship between the M3M and the IAC from panoramic radiographs [18]. Four hundred panoramic radiographs have been used to train the models with help of data augmentation [18]. Although all models showed high performance with the highest area under the receiver operating characteristic curve (AUC) values ranging from 0.88 to 0.93, the contact between the M3M and the IAC was not confirmed by CBCT in their study. This may lead to unexpected uncertainty in the model as many case studies showed that signs of contiguity on the panoramic radiograph did not always represent true contact between M3M and IAC [11,19,20]. This study also performed data augmentation, which is a common technique that help improve the generalizability and accuracy of the small dataset [21]. However, the study did not give an idea of how many numbers of the M3Ms should be augmented to achieve the best possible DL model.

To improve the reliability and accuracy of the panoramic radiographs used in model training, confirmation with CBCT images is desirable. In addition, it is commonly considered that DL model needs a large amount of data to train an efficient model. Although data augmentation is found to be beneficial to increase the number of data [21], designing augmented medical images that indeed preserved their labels may be computationally expensive and may take more time for the training process [22]. Therefore, the suitable number of augmented images should be studied. The aims of this study were to train DL models for automated recognition of contiguity between the M3M and the IAC from panoramic radiographs after confirming the proximity between those structures with CBCT images and to investigate the best effective fold of data augmentation to train a DL model.

## Materials and methods

All procedures were performed in accordance with the Declaration of Helsinki and the regulations approved by the Institutional Review Board from the Faculty of Dentistry/Faculty of Pharmacy, Mahidol University (CoA no. 2021/079.0109).

## Panoramic radiography and cone beam computed tomography (CBCT)

The panoramic radiographs were obtained from 1430 patients who were referred for the radiographic examination of the M3M at the Oral and Maxillofacial Radiology clinic, Faculty of Dentistry, Mahidol University, Thailand, between January 2013 to July 2021. These number of patients included those with only one M3M on the right or the left side of the mandible and those with two M3Ms, one on each side of the mandible. Among 1430 patients, 700 patients contained both panoramic radiographs and CBCT images. The panoramic radiographs were taken with a CS9000C machine (Carestream Health, Inc., New York, USA) with a tube voltage of 68–70 kV, tube current of 8–10 mA, and exposure time of 15.1 s. The CBCT images were taken with a 3D Accuitomo 170 machine (J Morita Mfg. Corp., Kyoto, Japan) with a tube voltage of 90 kV, tube current of 5 mA, and exposure time of 17.5 s. All the CBCT images were used regardless of the voxel sizes (i.e., 0.125 mm., 0.160 mm., and 0.250 mm.). The CBCT images were used to confirm the correlation between the risk signs on panoramic radiographs and direct contact on the CBCT images. The CBCT images must cover the entire root formation of the M3M and reveal its relationship to the IAC and be taken within 6 months of the panoramic radiographs.

## Classification of the M3M and IAC on panoramic radiographs

**Fig. 1:**
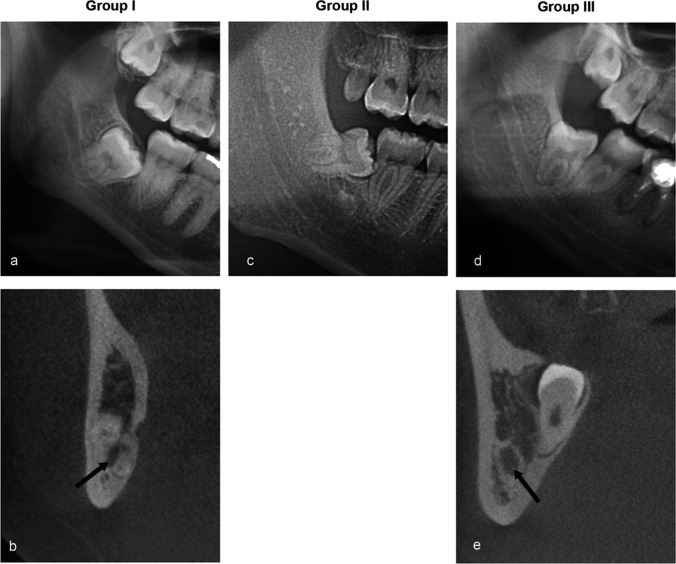
Examples of panoramic radiographs and CBCT images of mandibular third molars (M3Ms) used to evaluate the interrater and intrarater reliability. Panels **a** and **b** show a panoramic radiograph and a CBCT image in Group I, which illustrates direct contact between the M3M and the inferior alveolar canal (IAC). Panel **c** is a panoramic radiograph in Group II that clearly shows no contact. Panels **d** and **e** are a panoramic radiograph and a CBCT image from Group III, which shows sign of contiguity only on the panoramic radiograph. Arrows in panels **b** and **e** indicate the cross-sectional IAC

The panoramic radiographs were classified into three groups according to the presence of contiguity between the M3M and the IAC. Group I represented direct contact of the M3M on both panoramic radiographs and CBCT images. This group showed at least one risk sign on panoramic radiographs and no trabecular bone or marrow space between the M3M and the IAC on CBCT images (Fig. 1a, Fig. 1b). Group II clearly presented trabecular bone or marrow space between the M3M and the IAC on panoramic radiographs (Fig. 1c). Thus, it is not necessary to use CBCT images to confirm no contact in Group II. Group III is for false contact, which showed sign of contiguity on panoramic radiographs but no contact on CBCT images (Fig. 1d, Fig. 1e).

## Reliability test for group labelling

The group classification mentioned in the previous section was performed by D.P., a postgraduate student in oral and maxillofacial radiology. The reliability of data labelling was evaluated by cross-classification between D.P. and W.W., the oral and maxillofacial radiologist with 14-year experience. Ninety radiographs used in cross-classification were selected randomly and equally for each group by S.P., an oral and maxillofacial radiologist with 25-year experience. D.P. and W.W. independently carried out the classification twice in the two-week interval on the identical monitor with a resolution of 2560 × 1600 under dim light. The interrater and intrarater reliability showed perfect agreement with Cohen’s kappa of *κ* = 1.0 in both cases.

## Collection of panoramic radiographs

The inclusion criteria were the panoramic radiographs that presented the complete root formation of the M3M and revealed its relationship to the IAC. The exclusion criteria were the panoramic radiographs showing abnormalities or artefacts in the targeting region, showing an untraceable border of IAC at the area of M3M, and those showing false contact in Group III due to a very limited number of images. We began with studying 700 patients which contained both panoramic radiographs and CBCT images. We were able to include the total of 900 M3Ms in Group I—direct contact on both panoramic radiographs and CBCT images. This was the maximum number of M3Ms that could be collected according to the criteria of Group I. Then, we randomly selected an equal number of 900 M3Ms for Group II—no contact on panoramic radiographs. As a result, we have a final sample of 1800 M3Ms included in Groups I and II.

## Preparation of image sets

**Fig. 2:**
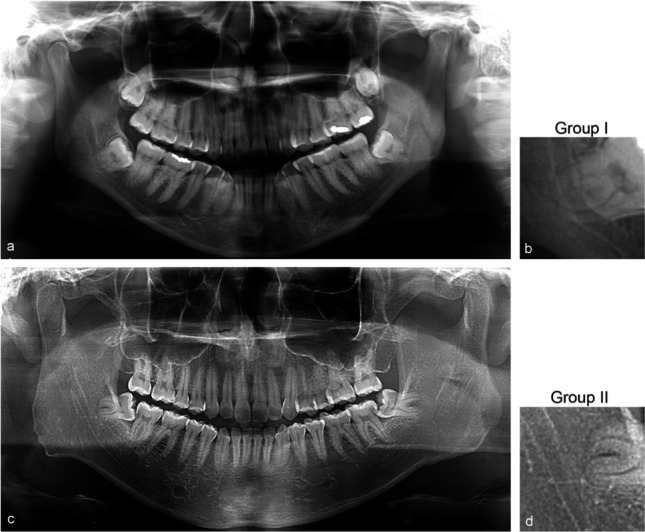
Image preparation. Left (a and c) and right (b and d) panels show the panoramic radiographs and 70 × 70-pixel cropped images centering at root apices between the mandibular third molar and IAC. Top panels (a and b) show an example from Group I, while the bottom panels (c and d) represent Group II

The radiographs were exported to Joint Photographic Experts Group (JPEG) format and cropped to the region of interest following the methods by Fukuda et al. [18]. The radiographs were exported with the highest quality setting of 1024 × 518 pixels and manually cropped using the Fiji program (Fiji, RRID:SCR_002285) [23]. Figure 2 shows the panoramic radiographs, which were manually cropped, mimicking the area that dentists focus on when they interpret these structures, into 70 × 70 pixels image patches centering at root apices of the M3Ms and IAC [18]. The 900 image patches in each group were randomly separated into three sets of 720, 90, and 90 images for the training, validation, and test sets, respectively [24].

## Training deep learning models

We used a transfer learning technique where pretrained CNNs were used as the starting point for our models [25]. Three highly acknowledged pretrained CNNs, AlexNet, VGG-16, and GoogLeNet, were used for training. AlexNet is a deep CNN that won the ImageNet Large Scale Visual Recognition Challenge (ILSVRC) in 2012 for its profound ability in natural image classification [26]. VGG-16 is a deep CNN developed in 2014 and well-known for the uniformity of its architecture. VGG-16 was designed based on the same principles as AlexNet but with more layers [27]. In other words, AlexNet contains 8 layers, while there are 16 layers in VGG-16. GoogLeNet, also known as Inception, was the winner model of the ILSVRC in 2014. Although it consisted of 22 layers deeper than previous models, it was designed to reduce a great number of parameters that help reduce the computational cost [28].

The pretrained CNNs were trained by using the FastAi library on Google Colaboratory [29]. FastAi is a high-level DL library built on PyTorch. It provides several sets of codes that practitioners can implement in various fields. Google Colaboratory is an online coding platform that allows writing and executing codes through a web browser. It also provides access to computational resources, including cloud graphic processing units (GPU).

**Fig. 3:**
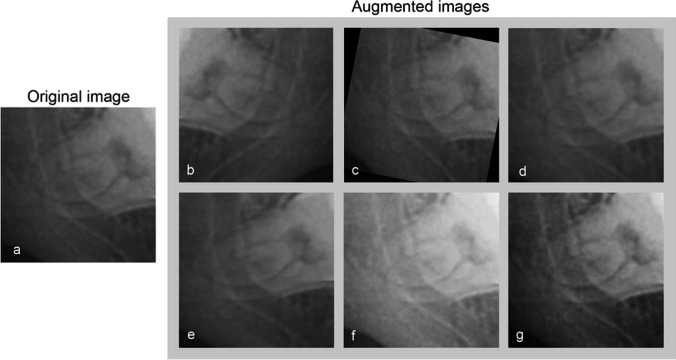
Examples of data augmentation techniques. The original image is shown in panel **a**. Panels **b–g** demonstrate augmentation techniques using horizontal flip, rotation, magnification, warp, brightness adjustment, and contrast adjustment, respectively

The training processes were divided into 2 rounds with only original images and with a data augmentation technique. We began the first round by training each of the three pretrained models with 1440 original image patches from the training sets; i.e., 720 images each for groups I and II. The learning rate was set at the default value of 0.001 for 1000 epochs. During training, the optimal number of iterations was selected by evaluating the accuracy from the prediction of 180 (90 × 2) image patches in the validation sets. The model was saved when the accuracy reached the optimum value with no improvement in the following twenty training iterations. The second training round was carried out with data augmentation to increase the number of image patches in the training data sets. Image augmentation techniques included horizontal flip, rotation, magnification, warp, brightness, and contrast adjustment (Fig. 3). All mentioned augmentation techniques were applied on every original image patch through FastAi library. We randomly selected image patches from each augmentation technique and created four new training sets: fivefold (7,200 images), tenfold (14,400 images), 15-fold (21,600 images), and 20-fold (28,800 images). The number of training images in parentheses also included the 1440 original images.

## Assessment of the diagnostic performance

The diagnostic performance of each trained model was evaluated by 180 (90 × 2) image patches in the test set. We defined the contact between M3M and IAC as positive, whereas no contact is negative. The receiver operating characteristic (ROC) curves were constructed, and the threshold of the prediction values for each model was determined by the Youden index [30]. Sensitivity, specificity, and accuracy were calculated at these thresholds. The summary of prediction results was presented in confusion matrices consisting of true positive (TP), false negative (FN), false positive (FP), and true negative (TN). The area under the ROC curve (AUC) was used as the main metric to evaluate the overall classifier performances of the models.

The statistical analyses were performed with MedCalc Statistical Software version 20.110 (MedCalc, RRID:SCR_015044). A comparison of the AUC among the models trained with only original images and among the models trained from the same pretrained models was performed by using the method of DeLong et al. (1988) [31].

## Results

**Fig. 4:**
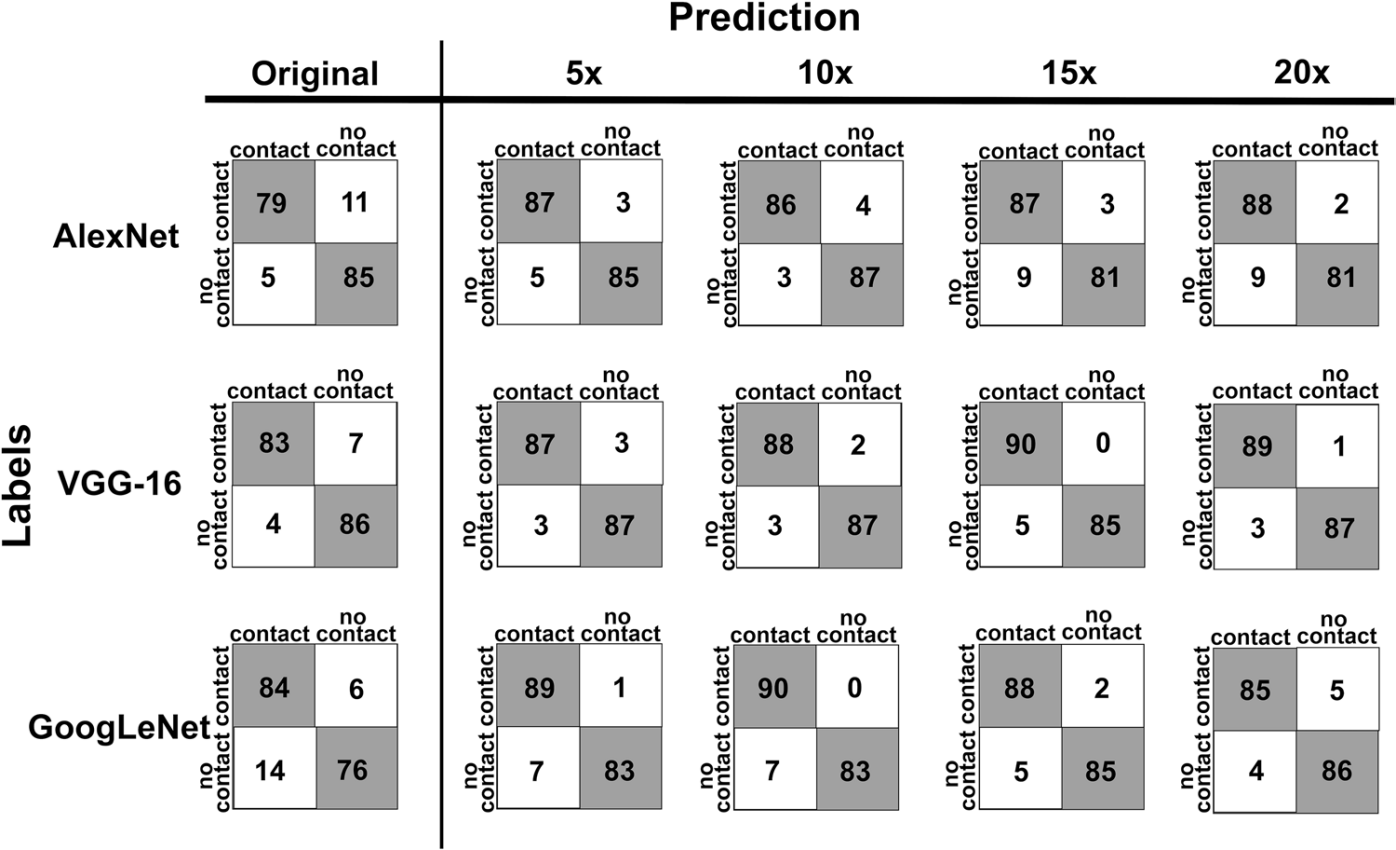
Confusion matrices of all 15 models. The Top, middle, and bottom rows are for AlexNet, VGG-16, and GoogLeNet. Columns from left to right are confusion matrices of the model with the original data set and the models with increasing numbers of trained images, including fivefold (5 ×), tenfold (10 ×), 15-fold (15 ×), and 20-fold (20 ×) increase. True positive, false negative, false positive, and true negative are shown in the top left, top right, bottom left, and bottom right cells of each matrix, respectively

Figure 4 shows the confusion matrices of all 15 models. As mentioned above, we have trained 3 pretrained models with each set of data, and we have one original data set and 4 sets of augmented data. The number of models is thus 15. The confusion matrix presented four values: TP, FN, FP, and TN. In general, all models correctly classified most of the test images into Group I (positive) or Group II (negative), as shown by the high TP and TN values. It is crucial not to misidentify Group I due to its higher risk of nerve injury; therefore, a low FN value is preferable. Based on the importance in predicting Group I correctly, VGG-16 predicted the least overall wrong images (FN + FP = 11) compared to AlexNet (FN + FP = 16), and GoogLeNet (FN + FP = 20). With data augmentation, the wrong predictions in Group I (FN) decrease significantly in all models, while it is marginal in the case of Group II wrong prediction (FP).

Table 1 shows the sensitivity, specificity, and accuracy. The model with high sensitivity is good at identifying most of the contact cases correctly, which is essential in identifying the patient who potentially has a harmful situation of the M3M and the IAC being contiguous. The high-specificity model is good at avoiding false recognition of no-contact cases. The model with high accuracy correctly classifies most of the cases to the correct group.

**Table 1:** Diagnostic performances of all 15 models

Models		Sensitivity (%)	Specificity (%)	Accuracy (%)	AUC^a^	*P*-value^b^
Pretrained CNN	Training set^c^					
AlexNet	Original	87.78	94.44	91.11	0.956	-
	5 ×	96.67	94.44	95.56	0.994	0.0174*
	10 ×	95.56	96.67	96.11	0.995	0.0152*
	15 ×	96.67	90.00	93.33	0.989	0.0343*
	20 ×	97.78	90.00	93.89	0.988	0.0386*
VGG-16	Original	92.22	95.56	93.89	0.981	-
	5 ×	96.67	96.67	96.67	0.990	0.3138
	10 ×	97.78	96.67	97.22	0.996	0.0817
	15 ×	100	94.44	97.22	0.992	0.2078
	20 ×	98.89	96.67	97.78	0.992	0.2113
GoogLeNet	Original	93.33	84.44	88.89	0.951	-
	5 ×	98.89	92.22	95.56	0.982	0.0151*
	10 ×	100	92.22	96.11	0.990	0.0091*
	15 ×	97.78	94.44	96.11	0.987	0.0026*
	20 ×	94.44	95.56	95.00	0.985	0.0040*

^a^*AUC*, area under the receiver operating characteristic curve

^b^*P*-value from AUC comparison with original training set within a pretrained CNN

^c^5 × , 10 × , 15 × , 20 × are fivefold, tenfold, 15-fold, and 20-fold augmented data, respectively

*P < 0.05

## Performance comparison among pretrained models with an identical number of data

**Fig. 5:**
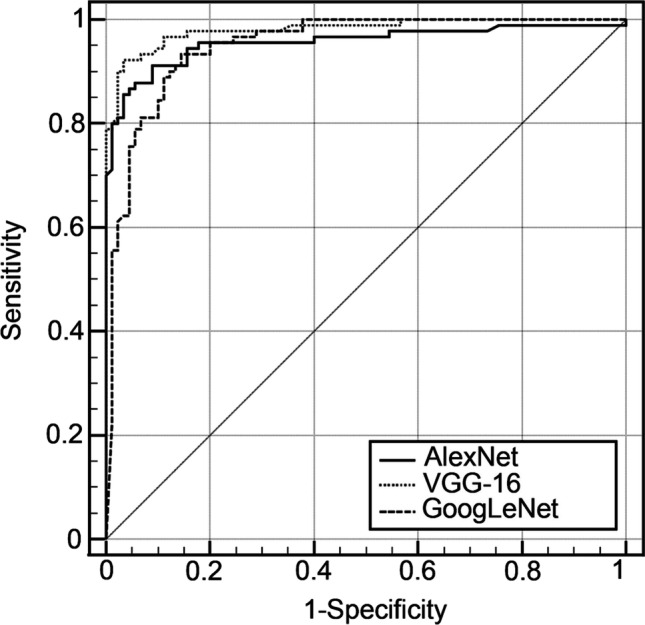
Receiver operating characteristic (ROC) curves of three different models trained with only original images. Solid, dotted, and dashed lines represent ROC curves of AlexNet, VGG-16, and GoogLeNet, respectively

Among the three models trained with the 1440 original images of M3Ms, VGG-16 showed the best overall performance with the highest AUC of 0.981 followed by AlexNet and GoogLeNet with AUC of 0.956 and 0.951, respectively (Table 1). The statistical analysis with MedCalc showed that the AUC of the VGG-16 model was better than that of GoogLeNet significantly with *P* < 0.05. However, there was no significant difference in AUC values between VGG-16 and AlexNet (AUC_VGG-16_: 0.981 versus AUC_AlexNet_: 0.956, *P* = 0.0574) and between AlexNet and GoogLeNet (AUC_AlexNet_: 0.956 versus AUC_GoogLeNet_: 0.951, *P* = 0.7832). Similarly, the ROC curves shown in Fig. 5 suggest that the VGG-16 model has the greatest discriminate capacity among the three models, followed by AlexNet and GoogLeNet, respectively.

When comparing AlexNet, VGG-16, and GoogLeNet trained with the same number of augmented images, we found that VGG-16 has the highest AUC values among the three models in all cases but fivefold, where AlexNet has a bit higher AUC (Table 1). However, there are no statistical differences in AUC values among the AlexNet, VGG-16, and GoogLeNet models.

## Performance comparison within pretrained models with different folds of data augmentation

**Fig. 6:**
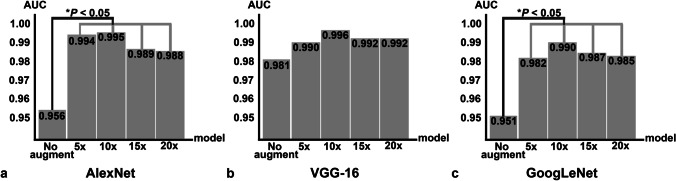
Bar graphs showing the area under the ROC curves (AUC) of models trained with five different data sets. Panels **a**, **b**, and **c** are the results of AlexNet, VGG-16, and GoogLeNet, respectively. In each panel, the left-to-right bar graph shows the AUC of models trained with the original data set and with the increasing numbers of trained images. Asterisks in panels **a** and **c** indicate the statistically significant differences between the AUC of the models trained with the original images and those with augmented images with *P* < 0.05

The AUC comparison of the models trained to distinguish between Group I and Group II is shown in Fig. 6. Within a pretrained CNN, the model trained with data set ten times larger than the original one showed the highest AUC, although the AUC values are not significantly different from the models trained with different numbers of augmented data. In contrast, if we compare the performance of models trained with only original images and with augmented images in different folds, the performance of AlexNet and GoogLeNet models improved significantly with *P* < 0.05 when increasing the number of images with data augmentation (AlexNet; AUC_AlexNet_: 0.956 versus AUC_AlexNet 5x_: 0.994, AUC_AlexNet_: 0.956 versus AUC_AlexNet 10x_: 0.995, AUC_AlexNet_: 0.956 versus AUC_AlexNet 15x_: 0.989, AUC_AlexNet_: 0.956 versus AUC_AlexNet 20x_: 0.988 (Fig. 6a, Table 1), and GoogLeNet; AUC_GoogLeNet_: 0.951 versus AUC_GoogLeNet 5x_: 0.982, AUC_GoogLeNet_: 0.951 versus AUC_GoogLeNet 10x_: 0.990, AUC_GoogLeNet_: 0.951 versus AUC_GoogLeNet 15x_: 0.987, AUC_GoogLeNet_: 0.951 versus AUC_GoogLeNet 20x_: 0.985 (Fig. 6c, Table 1)). However, increasing the number of images did not affect the performance of VGG-16 models, as shown in Fig. 6b and Table 1.

## Discussion

One aim of this study was to train DL models to recognize contiguity between the M3M and the IAC from panoramic radiographs. We selected not only one specific model but three different pretrained CNNs. All three models that are AlexNet, VGG-16, and GoogLeNet have shown good diagnostic performances as seen by the high sensitivity, specificity, accuracy, and AUC values. This study also supports the promising potential of DL models in evaluating the M3Ms and the IAC, similar to the previous studies [17,18,32,33].

We focused on preparing a highly accurate data. We assessed the relationship between the M3M and the IAC from CBCT images in every case in Group I to confirm our labels. We also collected up to 1800 original cropped images of M3Ms. This represents an excellent variety of M3Ms on panoramic radiographs; for instance, variation of the position or shape of the M3M, the various relationships between the M3M and the IAC, different densities of the surrounding bone, or various brightness or contrast of the radiographs. The accuracy of our labels and the great number and variety of our original images may lead to the higher AUC of the models when compared with the previous study [18]. Thus, to train an efficient DL model for medical image classification, the variety of the original data collected from the patients and the accurate labels annotated by the medical experts are crucial [24].

With only original data, we obtained the highest accuracy with the VGG-16 model, whereas AlexNet and GoogLeNet showed comparable accuracy (Fig. 6). As seen in Fig. 5, the ROC curves of AlexNet and VGG-16 showed a comparable ability to recognize the contiguity between the M3M and IAC. This is probably due to the similar concept of both models. VGG-16 improved the performance by increasing depth with multiple smaller-size (3 × 3) convolutional filters [27]. Meanwhile, GoogLeNet introduced the inception network where different sizes of convolutional filters are placed at the same level [28]. Kernels with different sizes are suitable and very effective for images containing various sizes of features. However, GoogLeNet could not give the best performance among the three pretrained models for our data set (Table 1). GoogLeNet may be more suitable for images with more details, unlike our cropped panoramic radiographs. Our results may suggest that the selection of the pretrained CNN can be determined by the nature of the data [34].

This is one of the first studies to investigate the effect of different folds of data augmentation on the diagnostic performance of the DL model in evaluating the M3Ms. We also compared the diagnostic performance of the models with those trained with the original data set. Although there was no significant difference compared with other fold of data augmentation, all three pretrained models showed the highest AUC values by training with tenfold (10 ×) data augmentation. Therefore, 10 × increase in the number of training data could possibly create an efficient DL model for a classification task involving M3Ms.

Interestingly, using only several original images with accurate labels could train an efficient DL model. Data augmentation obviously enhances the models’ performances since AlexNet and GoogLeNet were significantly improved when increasing the number and variety of images, even with fivefold data augmentation. However, the performance of VGG-16 trained without data augmentation was comparable with those using data augmentation. This suggested that either the number of our original images with accurate labels has already represented a significant variety of data to enhance models’ performances, or the number of original images is already saturated for VGG-16.

We have collected the panoramic radiographs from all patients who were also referred for CBCT to evaluate the M3M region from our database; however, it may not represent the optimum sample size for deep learning models. A recent systemic review emphasized the paucity of studies that determined the appropriate size of the training set for DL models and suggested the need for more studies focusing on the sample sizes [35]. The use of proper techniques for data augmentation showed promising potential in training well−performed models ^[[[22,36,37]]]^. However, from our result, it may not always be necessary. Further investigation of the optimum number of images necessary for training models should be conducted in the future study.

One limitation of this study is that we only used retrospective data from a single institution in both training and testing. Therefore, a prospective study using original images from various sources should be conducted. Moreover, federated learning could promote using multi-institutional data by sharing the model’s parameter trained from individual data instead of sharing the patient data directly [38,39].

Excluding all the radiographs showing abnormalities, artefacts, or those showing an unclear relationship between the M3M and IAC may affect the model’s performance in real-world clinical settings. We also excluded the panoramic radiographs that showed false contact (Group III); however, recognizing these radiographs would benefit clinical settings since it is difficult to notice even by experienced observers. We decided to exclude the panoramic radiographs in Group III, showing false contact, because a very limited number of images (fewer than 100 cases) could be obtained. When training with only original images, including Group III could possibly create bias from a class imbalance that favors the prediction of Group I and II [40,41]. Our further study will certainly focus more on these excluded radiographs, especially those in Group III, to make the clinical application of the model possible.

The validation sets splitting remained constant across the training process of all models. The test set also remained constant for the evaluation of the models’ performances [42]. This eliminated the difference in the performance among models that may be caused by the variability in the validation set and test set. However, the ratio of the number of training images to the validation images was different among five conditions (no augmentation, fivefold, tenfold, 15-fold, and 20-fold). This may have affected the results of our study and can be considered as another limitation of our study [43].

There are many prospects to develop our models. Training the models with only images indicated that the models considered only information from pixel data. However, most practitioners evaluate the radiograph along with considering other clinical information for a better diagnosis. DL models, similar to practitioners, also learn better with more information provided [44,45]. Additional clinical information could be given with the radiographs in the training process.

We also consider developing an end-to-end learning model as a final product that could automatically classify the panoramic radiographs in the DICOM file from the machine. We will further investigate whether cropping panoramic radiographs necessarily improves the model’s performance. Additional data on the bucco-lingual relationship between the M3M and IAC will also be collected to improve the clinical usefulness of the model for surgical planning. We aim to further develop the model as an assistant diagnostic tool to help confirm the dentists’ decision on whether to refer the case for CBCT investigation, particularly the referral of patients from a rural hospital to a provincial hospital. This confirmation can help assure that the referral does not unnecessarily cost the patient time and money.

## Conclusion

All 15 models showed good performance in recognizing contiguity between the M3M and IAC on panoramic radiographs with the AUC ranging from 0.951 to 0.996. Within pretrained models, a 10 × of training data augmentation showed the highest AUC values in all three models. However, there were no significant difference when compared with fivefold, 15-fold, and 20-fold data augmentation. Among pretrained models, VGG-16 mainly showed the best performance when training with the same fold of data augmentation. VGG-16 also showed the best performance among models trained only with 1440 (720 × 2) original images and showed comparable results with and without data augmentation. A variety of the original images with accurate labels may be sufficient to train a well-performed model using VGG-16.
